# Managing Symptom Profile of IBS-D Patients With Tritordeum-Based Foods: Results From a Pilot Study

**DOI:** 10.3389/fnut.2022.797192

**Published:** 2022-02-15

**Authors:** Francesco Russo, Giuseppe Riezzo, Michele Linsalata, Antonella Orlando, Valeria Tutino, Laura Prospero, Benedetta D'Attoma, Gianluigi Giannelli

**Affiliations:** ^1^Laboratory of Nutritional Pathophysiology, National Institute of Gastroenterology, IRCCS “Saverio de Bellis”, Castellana Grotte, Bari, Italy; ^2^Laboratory of Nutritional Biochemistry, National Institute of Gastroenterology, IRCCS “Saverio de Bellis”, Castellana Grotte, Bari, Italy; ^3^Scientific Direction, National Institute of Gastroenterology, IRCCS “Saverio de Bellis”, Castellana Grotte, Bari, Italy

**Keywords:** diet, dysbiosis, gastrointestinal symptoms, inflammation, intestinal permeability, irritable bowel syndrome, Tritordeum

## Abstract

In the past few years, increasing attention has been given to the pathologic role of specific foods in IBS, like wheat and other cereals. Recent literature describes IBS patients who may experience gastrointestinal (GI) and extra-GI symptoms precipitated by the ingestion of cereals. Tritordeum is a cereal of Spanish origin derived from the hybridization of durum wheat and wild barley. It is different from classic wheat for its gluten protein composition, with fewer carbohydrates and fructans and a higher content of proteins, dietary fibers, and antioxidants. This pilot study aimed to investigate the effects of a 12-week diet with Tritordeum-based foods in substitution of other cereals on the profile of GI symptoms (evaluated by appropriate questionnaire) and the health of the GI barrier (assessed by sugar absorption test and different markers of integrity and functions) in 16 diarrhea-predominant IBS (IBS-D) patients. The diet with Tritordeum-based foods (bread, bakery products, and pasta) significantly reduced IBS-D patients' symptoms. This amelioration appears to occur through an overall improvement of the GI barrier, as demonstrated by the reduced intestinal permeability and the decreased levels of markers of intestinal mucosal integrity, mucosal inflammation, and fermentative dysbiosis.

## Introduction

About 10–15% of the adult population worldwide is affected by irritable bowel syndrome (IBS), a functional, non-organic gastrointestinal disorder (FGID) that significantly impairs the quality of life (1). Since there are still no reliable biomarkers, IBS is defined by the presence of characteristic symptoms (mainly recurrent abdominal pain or discomfort with modifications in the stool habit) described by Rome criteria (2). Furthermore, IBS patients can be categorized into different IBS subgroups based on the predominant bowel pattern (IBS-D: defecation with diarrhea; IBS-C: defecation with constipation; IBS-M: a mixture of diarrhea and constipation) (3).

Many IBS patients often describe a worsened symptom profile in consuming foods containing the so-called fermentable oligosaccharides, disaccharides, monosaccharides, and polyols (FODMAPs). FODMAPs are present in certain foods, including wheat and beans, and may contribute to digestive symptoms like abdominal pain, bloating, and diarrhea since the small intestine poorly absorbs them. In such a way, they are digested by colonic bacteria, releasing gas in the intestine. In addition, FODMAPs can exert an osmotic effect increasing the volume of water in the stool (4).

Thus, alternative nutritional approaches, such as the low FODMAP diet (LFD), have been evaluated, proving helpful for treating IBS. In recent studies by our group, IBS-D patients benefited from a long-lasting (12 weeks) LFD in terms of symptoms, inflammatory status, vitamin D content, and lipidomic profiles (5, 6). However, adherence to LFD is somewhat problematic, often requiring constant nutritional support.

In the past few years, increasing attention has been given to the pathologic role of specific foods in IBS, like wheat and other cereals. Recent literature describes IBS patients who may experience gastrointestinal (GI) and extra-GI symptoms precipitated by the ingestion of cereals. The main culprit in this chain of pathophysiological events is gluten, and the spectrum of gluten-related disorders, apart from celiac disease (CD), includes herpetiform dermatitis, gluten ataxia, wheat allergy, and non-celiac wheat sensitivity (NCWS) (4–6). However, the complex milieu of nutrients in wheat-based foods, other than gluten, could be responsible for the multifaceted manifestations of IBS-like symptoms.

While in the CD pathogenesis, the immune response against the prolamins of cereals involves innate and adaptive immunity, the innate immunity alone seems to be responsible for NCWS (7). In this framework, it has been hypothesized that some monococcal diploid grain lines, characterized by a minimal activation of the innate immunity and a reduced amount of toxic gluten peptides, could be used in the diet of NCWS patients. This hypothesis deserves further clinical investigation and could be relevant also for other GI conditions, mainly IBS. Patients with IBS-D and IBS-M who generally suffer from abdominal bloating as the dominant symptom seem to benefit from eliminating wheat from the diet (8). A gluten-containing diet alters intestinal barrier function in IBS-D patients, and a previous study by Aziz et al. (9) demonstrated that a 6-week gluten free diet (GFD) reduced the score of the IBS Symptom Severity Score (IBS-SSS) by ≥50 points in 71% of the cases, and this finding was independent of their HLA-DQ2/8 genotype. Thus, eliminating gluten from the diet could be beneficial also for these subjects.

Tritordeum is a cereal of Spanish origin derived from the hybridization of durum wheat and wild barley. It has already grown in Spain and Portugal and, more recently, in Apulia, a southern Italy region. The cultivation of this cereal is based on traditional techniques, and it grows well with little care, being resistant to drought, heat, and disease (10). Tritordeum has the unique characteristic of having a protein composition of gluten different from that of classic wheat, with significantly lower levels of gliadins, fewer carbohydrates and fructans, and a higher content of proteins, dietary fibers, and antioxidants (11). Although Tritordeum is not suitable for CD patients as it still contains gluten, it could be used for producing foods for patients with NCWS and those with IBS-D or IBS-M (12).

Many of the above-cited patients may show an altered epithelial barrier in the gut mucosa with changes in the small intestinal permeability (s-IP) (13). It is now established that a dysfunctional intestinal barrier could be tightly linked to persistent low-grade immune activation, thus playing a critical role in IBS (14). The evaluation of s-IP is based on the assessment of the urinary recovery of orally administered non-absorbable sugars of different sizes [e.g., lactulose (La), mannitol (Ma), and sucrose (Su)] (15). La is a disaccharide that gives information on the integrity of the paracellular pathway and tight junction (TJ), while Ma is a monosaccharide, reflecting the transcellular route. Thus, the urinary La/Ma ratio is used to evaluate the conditions of s-IP. The third probe, Su, is an index of gastro-duodenal permeability (16). The GI barrier function is also investigated by assaying the zonulin levels in serum and feces, a human protein that controls s-IP by changing the TJ interactions (17). The serum circulating levels of intestinal fatty acid-binding protein (I-FABP) (18) and diamine oxidase (DAO) (19) are now considered as potential markers for the intestinal epithelial barrier health since they are immediately released in response to the cell membrane's altered integrity (20).

Based on these premises, and in line with our previous research on possible different nutritional approaches as treating options for IBS-D patients (5, 6), the present pilot study aimed to investigate the effects of administering for 12 weeks Tritordeum-based foods (bread, bakery products, and pasta) instead of the corresponding wheat-containing products on the symptom profile and GI barrier function of IBS-D patients.

The GI symptoms were assessed by the IBS-SSS (21) and the state of health of the GI barrier by evaluating the above-mentioned urinary and circulating markers. The minimal inflammation was assessed by dosing interleukins 6 and 8 (IL-6 and IL-8). Lastly, lipopolysaccharide (LPS) and the urinary markers of intestinal dysbiosis (indican and skatole) were also assayed.

## Materials and Methods

### Patient Recruitment

The inclusion criteria for this pilot study were a diagnosis of IBS-D according to the Rome IV criteria and age 18–65 years. Patients were recruited for this study from January 2018 to September 2020 among the Nutritional Physiopathology Laboratory's outpatients—National Institute of Gastroenterology “S. de Bellis” Research Hospital.

The exclusion criteria were GI symptoms with a total score lower than 125 on IBS-SSS (21), presence of any organic GI disease, severe organic and psychiatric diseases, or already on a diet (i.e., vegan diet, gluten-free diet, low carbohydrate high-fat diet, or LFD). In the case of lactose intolerance, the lactose-reduced diet was allowed as long as patients agreed to keep this intake constant during the study period. Patients also had to be willing to change their current diet to enter the study.

All patients received study-specific verbal and written information before giving their written consent.

At baseline, celiac-specific serum IgA and IgG titers against tissue transglutaminase and endomysium were evaluated in all the recruited subjects to exclude CD. Moreover, only those patients who resulted negative/negative in the HLA-DQ2/HLA-DQ8 determination were included in the study to avoid symptoms due to NCWS complained by some IBS patients positive for HLA-DQ2 or DQ8 (22).

Total and wheat-specific immuno-globulin E antibodies were determined in all participants before starting the study (ImmunoCAP™250, ThermoFisher Scientific, Germany) to exclude wheat allergy. Besides, a prick test was requested to exclude cutaneous wheat sensitization.

The present study is part of broader research comparing alternative nutritional strategy based on foods with low FODMAP content respect to usual dietary advice administered to the IBS-D patients. The study received the approval of the Scientific Committee of IRCCS “S. de Bellis” and the Ethics Committee of IRCCS “Ospedale Oncologico – Istituto Tumori Giovanni Paolo II”, Bari, Italy (N. 274/C.E. 12.12.17). The trial has been registered on http://www.clinicaltrials.gov---NCT03423069.

All the subjects in the study gave their written informed consent to collect anthropometric and analytical measurements and clinical data.

### Study Design

**Figure 1:**
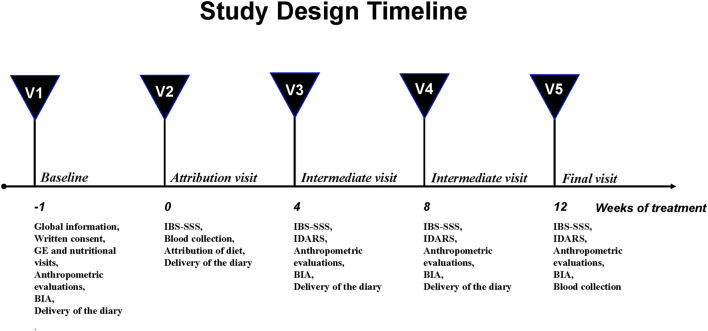
Study design.

The study design was structured on five visits and has already been described elsewhere (5) (Figure 1).

Briefly, at baseline (V1), the subjects underwent a physical examination by a gastroenterologist and an interview with a nutritionist. The anthropometric measurements and the bioelectrical impedance analysis (BIA) were performed. The eligible patients consumed their habitual diet and filled in a daily diary of their food intake and their intestinal habits according to the Bristol stool form chart (23). Besides, their physical activity and the use of drugs were recorded to precisely assess their daily intake and energy consumption.

One week after visit 1 (V2), all the patients completed the IBS-SSS questionnaire (21) and had to get a total IBS-SSS score >125 to be recruited. Then, the eligible subjects had a controlled diet, and they were also invited to fill the daily diary until the end of the diet. Besides, they supplied stool and urine samples and underwent a blood sample withdrawal and the sugar absorption test (SAT).

During the intermediate control visits (V3, after 4 weeks and V4, after 8 weeks), the symptom and food questionnaires completed in the weeks before were collected, and the patients received the new IBS-SSS and the questionnaire on adherence to the diet (IBS Diet Adherence Report Scale—IDARS). This questionnaire consists of five questions on adherence to dietary treatment, with a score for each item ranging from one to five. A total score equal to or higher than 20 represents good adherence to the diet (24). BIA and anthropometric measurements were also performed.

After 12 weeks of the assigned diet (V5), the researchers collected the symptom and food questionnaires filled in the preceding weeks. The patients compiled the IBS-SSS and a food diary to check their adherence to the proposed diet (IDARS) and underwent the same procedures as visit 2 for the clinical, anthropometric and analytical measurements.

### Symptom Profile

The symptom profile was evaluated using a validated questionnaire for GI symptoms, the IBS-SSS (21). This questionnaire gives a global measure of symptoms' severity by evaluating five items (namely, “severity of abdominal pain,” “frequency of abdominal pain,” “severity of abdominal distension,” “dissatisfaction with bowel habits,” “impact of symptoms on quality of life”) on a visual analog scale.

Each symptom was graduated on a 100-point scale. For the first four items, patients marked a point on the line that represented how they felt, and the proportional distance from zero (ranging from 0 to 100) represented the score of that item. The fifth item required the number of days out of 10 during which the subjects complained of “abdominal pain.” The answer was multiplied by 10 to create a metric scale from 0 to 100. The five items were then added up to a total score between 0 and 500. Cases were so categorized as “mild” (75–175), “moderate” (175–300), and “severe” (>300). Healthy subjects conventionally have a score below 75, whereas patients with scores lower than 75 are considered in a remission phase.

### Assessment of Nutrient Intakes

Throughout the study, the patients consumed a controlled diet and abstained from alcohol or heavy physical activity. All dietary instructions were provided by trained staff, who interviewed each subject before the study to obtain a report of their usual diet and calculate the energy requirement (see V1 in Study Design). Patients recorded a food diary during the study to assess their energy intake and energy consumption.

The energy intake refers to the quantity of calories introduced with food and drinks in the unit of time (24 h). The energy consumption represents the total energy expenditure, spent by the body in the unit of time (24 h), needed by the organism to the maintenance of its structural and functional characteristics, and for physical activity.

The diary included the details of the quantities (expressed in grams) and the description of food consumed every day for breakfast, lunch, dinner, snacks, as well as physical activity and its duration (5, 6).

Nutritionists then evaluated the food diaries completed at the start of the study and during the diet. All data were evaluated by dedicated software (Progetto Dieta v. 2.0—http://www.progettodieta.it—last access date: March 18, 2020). The daily energy intake and consumption (in kcal), the carbohydrates, lipids, and proteins (expressed as percentage and weight), the consumption of alcohol (expressed as a percentage), and dietary fiber (in grams) were calculated.

### Tritordeum-Based Foods Characteristics

All the food items (bread, pasta, “taralli”—local salty biscuits, and breakfast biscuits) were prepared using Tritordeum flour. The energy content and chemical composition of Tritordeum flour and pasta are represented in Table 1.

**Table 1:** Energy content and chemical composition of Tritordeum flour and pasta.

**Energy per 100 g**	**Flour**	**Pasta**
Kcal	350	355.6
Proteins (g)	12.8	12
Fats (g)	1.7	0.1
Saturated fats (g)	0.4	0.0
Dietary fiber (g)	2.6	1.5
Carbohydrates (g)	69.6	75.9
Simple sugars (g)	0.8	4.3
Salt (g)	0.1	0.2

The Tritordeum pasta formulation was as follows: water and Tritordeum semolina. In terms of pasta shape, each formulation was made up of long pasta (spaghetti, linguine, and fettuccine) and short pasta (penne rigate and rigatoni) in a pilot pasta-making plant (Intini Food, Putignano—Bari, Italy).

The bread ingredients were: Tritordeum flour, water, salt, brewer's yeast. Once the dough was ready and risen sufficiently to reach double the volume, it was baked for about 30 min at 240°C.

The ingredients of taralli were Tritordeum flour, olive oil, white wine, salt. These salty biscuits were prepared by baking the ready dough for about 40 min at 250°C.

The breakfast biscuits were made with Tritordeum flour, lactose-free milk, olive oil, sugar, ammonium bicarbonate. Once the dough was ready, it was baked for about 20 min at 200°C.

### Intervention Diet

A controlled Tritordeum-based diet was provided to each patient. The daily menu was based on breakfast, mid-morning snacks, lunch, afternoon snacks, and dinner. This intervention diet implied that each patient in the study had to consume flour, bread, breakfast biscuits, taralli, and pasta prepared exclusively with Tritordeum.

The diets were prepared as described elsewhere (6) by matching basal metabolism and daily energy consumption with anthropometric data of all patients to assign satisfactory and tailored dietary regimens. An appropriate software (Nutrigeo 8.6.0.0, Progeo Medical, Centobuchi di Monteprandone, AP, Italy) was utilized to assess the daily intake of macronutrients (50% carbohydrates, 30% lipids, and 20% proteins). Every 30 days, patients had to undergo in-between visits, during which they had to write the IDARS.

### Sugar Absorption Test (SAT)

All the patients underwent SAT for s-IP evaluation after an overnight fast. After collecting pre-test urine to exclude the potential presence of endogenous sugars, the patients drank the test solution prepared with La (10 g), Ma (5 g), and Su (40 g) in a final volume of 100 mL and samples of urine were collected up to 5 h. The total urine volumes of each patient were evaluated and recorded. After mixing, 2 mL samples were taken and kept at −80°C until the examination.

The analysis of the three probes (La, Ma, and Su) in urine were performed by chromatographic methods, as described before (25). The percentage of administered La (%La), Ma (%Ma), and Su (%Su) were measured, and the La/Ma ratio was calculated for each sample. A La/Ma ratio higher than 0.03 represented altered s-IP (26).

### Biochemical Assays

All the analyses were conducted at the start and the end of the treatment. A whole blood sample was withdrawn and collected in vacutainer tubes containing ethylene–diamine–tetra-aceticacid anticoagulant from each patient after fasting overnight. Raw stool samples were collected, frozen, and kept at −80°C within 12 h after the sampling until the laboratory evaluation. Stool specimens were then thawed and homogenized by an inoculation loop. An appropriate kit for the preparation of fecal eluates was applied (Fecal Sample Preparation kit—Immunodiagnostik AG, Bensheim, Germany).

Zonulin was evaluated in serum and fecal samples by ELISA kits (Immunodiagnostik AG, Bensheim, Germany). According to the manufacturer's instructions, normal serum and fecal concentrations had to be below 48 and 107 ng/mL, respectively. I-FABP and DAO's serum levels were assessed by ELISA kits (Thermo Fisher Scientific, Waltham, MA, USA and Cloud-Clone Corp. Houston, TX, USA, respectively).

The serum levels of IL-6 and IL-8 were measured by ELISA kits (BD Biosciences, Milan, Italy). Lastly, LPS circulating concentrations were assayed by an ELISA kit (Cloud-Clone Corp. Katy, TX, USA).

### Indican and Skatole Evaluation

The evaluation of urinary indican was performed using a colorimetric assay kit (ABNova Corp., Taipei, Taiwan).

The urinary skatole was assayed by the 3-methylindole kit (EurekaLab Division, Chiaravalle, AN, Italy) on a Thermo Scientific model Dionex high-performance liquid chromatography (HPLC) system equipped with an UltiMate 3000 pump and a Rheodyne injector with a 20-μL loop (Sunnyvale, CA, USA) as previously described (5).

According to previous reports (27), urinary indican and skatole were considered normal at values lower than 10 mg/L and 10 μg/L, respectively. Urinary concentrations of indican and skatole higher than 20 mg/L and 20 μg/L indicate the presence of fermentative and putrefactive grade I dysbiosis, respectively (28).

### Statistics

No studies of Tritordeum in IBS have been performed previously, and the present research represents a pilot study. Thus, based on these premises, the statistical power calculation was not necessary. Statistics were performed using Sigma Stat 11.0 (Systat Software, Inc., San Jose, CA, USA) and GraphPad Prism 5 (GraphPad Software Inc., La Jolla, Ca, USA). A non-parametric test was performed to avoid the assumption of normal distribution, and because of the small number of patients studied, specifically Wilcoxon rank-sum test was used to compare pre- and post-treatment data. Unless otherwise specified, all results were expressed as means ± SEM. *P* < 0.05 was considered statistically significant.

## Results

### Number and Anthropometric Characteristics of the Patients

**Figure 2:**
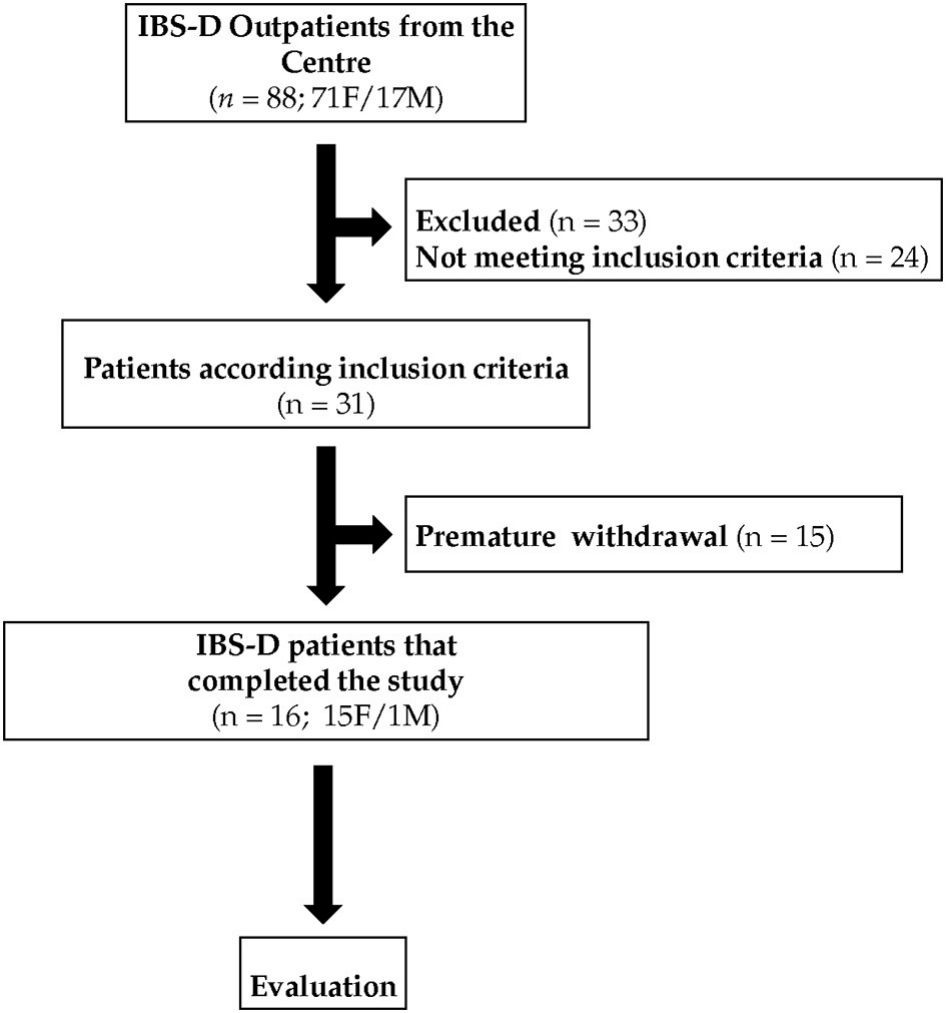
The flow of the patients through the study.

Figure 2 summarizes the flow of the patients through the study. Eighty-eight patients, 71 females (F) and 17 males (M) with IBS-D, were recruited. Of these patients, 24 did not meet the inclusion criteria, 33 were excluded for different reasons (pregnancy, abdominal surgery, use of antibiotics, moved to other districts, change of working activities), and 15 withdrew consent or were excluded due to dietary transgressions. Sixteen IBS-D (1M and 15 F) patients completed this pilot study following the diet for 12 weeks. During the study, all the participants excluded wheat from the diet completely.

Table 2 reports the patients' anthropometric characteristics before and after a Tritordeum-based diet. Compared to the study's start, significant decreases in weight, body mass index (BMI), abdominal and waist circumferences were observed at the end of the diet. Besides, as concerns BIA parameters, the Phase Angle (PhA) significantly increased (*P* < 0.05) whereas Fat-Free Mass (FFM), Total Body Water (TBW), and Extra Cellular Water (ECW) were significantly reduced (*P* < 0.05) after the diet in comparison with the diet's start.

**Table 2:** Anthropometric characteristics of the IBS-D patients before (Pre) and after (Post) 12 weeks of Tritordeum-based diet.

	**Pre (*n* = 16)**	**Post (*n* = 16)**	***P***
Age (years)	45.94 ± 2.68	–	–
Sex (M/F)	1/15	–	–
Weight (Kg)	65.81 ± 2.66	63.28 ± 2.59	0.002
Height (m)	1.60 ± 0.02	–	–
BMI (Kg/m2)	25.65 ± 1.19	24.64 ± 1.15	0.004
Abdominal circumference (cm)	91.49 ± 2.62	88.31 ± 2.55	0.038
Waist circumference (cm)	80.63 ± 3.11	78.01 ± 2.89	0.009
PhA (degrees)	5.86 ± 0.14	6.18 ± 0.10	0.005
BCM (Kg)	24.34 ± 0.99	24.94 ± 1.08	ns
FM (Kg)	19.83 ± 1.78	18.62 ± 1.61	ns
FFM (Kg)	46.16 ± 1.66	44.91 ± 1.57	0.017
TBW (liters)	33.66 ± 1.25	32.57 ± 1.18	0.032
ECW (liters)	15.62 ± 0.59	14.38 ± 0.51	0.004

*BMI, Body Mass Index; PhA, Phase Angle; BCM, Body Cell Mass; FM, Fat Mass; FFM, Fat-Free Mass; TBW, Total Body Water; ECW, Extra Cellular Water. Data are expressed as means ± SEM and analyzed by Wilcoxon signed-rank test. All differences were considered significant at P < 0.05*.

The patients' daily nutritional information recorded at the start and the end of the Tritordeum-based diet are shown in Table 3.

**Table 3:** Daily nutritional information of the IBS-D patients before (Pre) and after (Post) 12 weeks of Tritordeum-based diet.

	**Pre (*n* = 16)**	**Post (*n* = 16)**	***P***
Energy consumption (kcal)	2,147 ± 85.40	2,174 ± 86.57	Ns
Energy intake (kcal)	1,905 ± 155.70	1,513 ± 31.46	Ns
Basal metabolism (kcal)	1,456 ± 28.78	1,473 ± 31.36	ns
Proteins (g)	72.34 ± 4.85	75.63 ± 1.67	ns
Proteins (%)	17.04 ± 0.44	20.0 ± 0.16	0.0005
Lipids (g)	90.17 ± 10.03	50.42 ± 1.08	0.0006
Lipids (%)	41.33 ± 1.59	30.0 ± 0.16	0.0005
Carbohydrates (g)	191.50 ± 15.06	200.10 ± 4.62	ns
Carbohydrates (%)	41.12 ± 1.85	49.57 ± 0.20	0.0009
Alcohol (%)	0.71 ± 0.39	0.56 ± 0.22	ns
Dietary fiber (g)	14.50 ± 1.23	15.14 ± 0.39	ns

*Data are expressed as means ± SEM and analyzed by Wilcoxon signed-rank test. All differences were considered significant at P < 0.05*.

A significant reduction of lipid grams and the percentage was observed at the end of the diet compared to baseline. On the contrary, a significant increase in protein and carbohydrate percentage resulted after the Tritordeum-based diet compared to the start of the study.

### The Symptom Profile in IBS-D Patients

Table 4 reports the single items and total scores of the IBS-SSS questionnaire in the IBS-D patients before and after the diet. All the single items significantly decreased after 12 weeks of diet. Specifically, “Abdominal pain intensity” reduced by 44.9%, “Abdominal pain frequency” by 45.1%, “Abdominal distension” by 51.2%, “Dissatisfaction of bowel habit” by 45.5%, and “Interference on life in general” by 44.9%. Lastly, the “Total score” reduced by 46.4% after diet.

**Table 4:** Irritable bowel syndrome-symptom severity scale (IBS-SSS) single items and total score of IBS-D patients before (Pre) and after (Post) 12 weeks of Tritordeum-based diet.

	**Pre (*n* = 16)**	**Post (*n* = 16)**	***P***
Abdominal pain intensity	51.38 ± 5.93	28.31 ± 6.14	0.0020
Abdominal pain frequency	50.77 ± 9.09	27.85 ± 7.48	0.0110
Abdominal distension	65.38 ± 5.30	31.92 ± 6.40	0.0020
Dissatisfaction of bowel habit	77.69 ± 4.51	42.31 ± 6.29	0.0005
Interference on life in general	59.38 ± 7.17	32.69 ± 5.98	0.0010
Total score	304.60 ± 19.33	163.10 ± 31.26	0.0002

*Data are expressed as means ± SEM and analyzed by Wilcoxon signed-rank test. All differences were considered significant at P < 0.05*.

### The Small Intestinal Permeability (s-IP)

**Figure 3:**
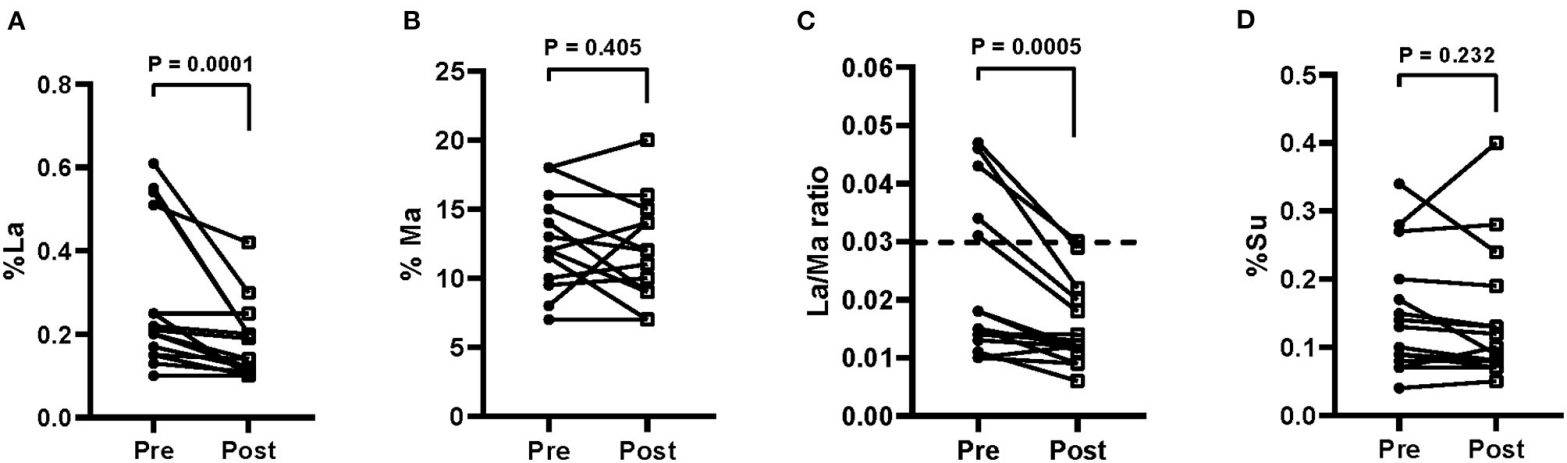
Small intestinal permeability as evaluated by the sugar absorption test in urine [**A** = % lactulose (La); **B** = % mannitol (Ma); **C** = La/Ma ratio; **D** = % sucrose (Su)] before (pre) and after (post) 12 weeks of Tritordeum-based diet in 16 IBS-D patients. Data expressed as means ± SEM. Wilcoxon rank-sum test was used to compare pre- and post-treatment data. Differences were considered significant at *P* < 0.05. Dotted line indicates the cut-off values for the La/Ma ratio. A La/Ma ratio higher than 0.03 represented altered s-IP.

The s-IP in IBS patients was evaluated by the SAT before the diet and at the end of treatment. Figure 3 shows the urinary percentages of the three probes %La, (Figure 3A) %Ma, (Figure 3B) and %Su (Figure 3D). The La/Ma ratio was estimated in all the samples (Figure 3C).

At the end of the diet, %La was significantly (*P* = 0.0001) lower than the baseline percentage (0.28 ± 0.04 vs. 0.18 ± 0.02), whereas %Ma was not different from starting value (13.0 ± 0.84 vs. 12.0 ± 0.89). The La/Ma ratio decreased significantly (*P* = 0.0005) (0.022 ± 0.003 vs. 0.015 ± 0.002). Finally, no reduction of %Su at the end of the diet occurred (0.15 ± 0.02 vs. 0.14 ± 0.02).

### Biomarkers of Intestinal Barrier Function and Integrity

**Figure 4:**
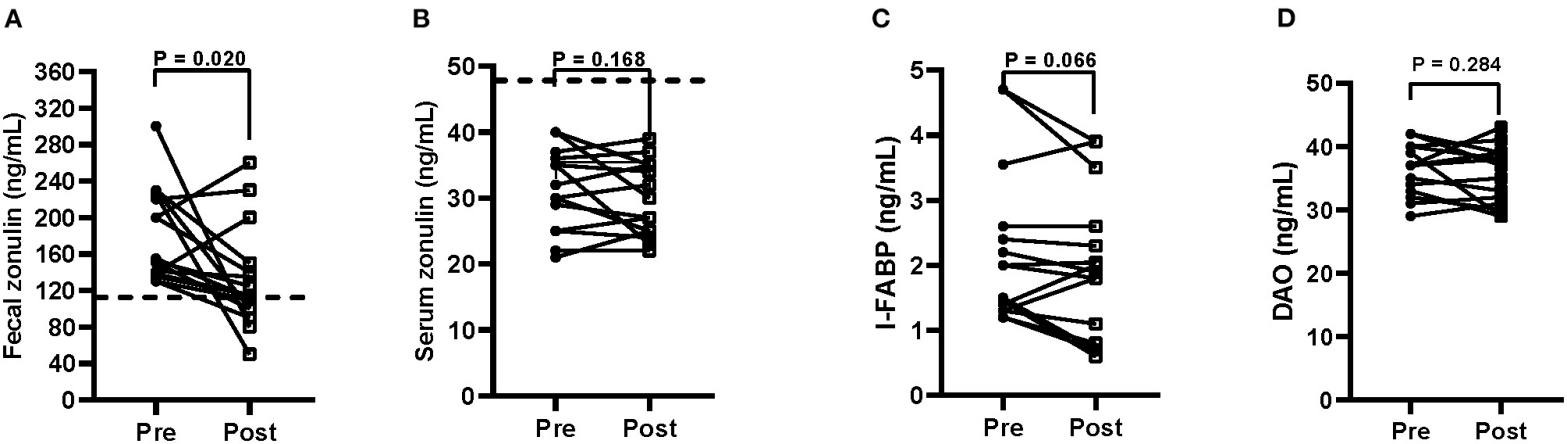
Biomarkers of intestinal barrier function and integrity (**A** = fecal zonulin; **B** = serum zonulin; **C** = serum intestinal fatty acid-binding protein—I-FABP; **D** = serum diamine oxidase—DAO) before (pre) and after (post) 12 weeks of Tritordeum-based diet in 16 IBS-D patients. Data expressed as means ± SEM. Wilcoxon rank-sum test was used to compare pre- and post-treatment data. Differences were considered significant at *P* < 0.05. Dotted line indicates the cut-off values for fecal and serum zonulin. Normal fecal and serum zonulin concentrations have to be below 107 and 48 ng/mL, respectively.

Figure 4 reports the fecal and serum zonulin concentrations (Figures 4A,B) and the serum levels of I-FABP (Figure 4C) and DAO (Figure 4D).

At the start of the study, mean fecal zonulin concentrations in patients with IBS-D were above the cut-off level (180.0 ± 13.1 ng/mL). After the diet, the fecal zonulin concentrations reduce significantly (*P* = 0.020) (132.1 ± 14.0 ng/mL), reaching values below the cut-off limit in 9 out 16 patients (56%). On the contrary, serum zonulin values were not affected by the diet (31.0 ± 1.5 vs. 29.1 ± 1.4 ng/mL). As for the integrity of the intestinal barrier, the serum I-FABP concentrations were not different between the start and the end of the study (2.2 ± 0.30 ng/mL vs. 1.9 ± 0.28 ng/mL). Similarly, serum DAO levels resulted unaffected by treatment (36.1 ± 0.97 ng/mL vs. 35.0 ± 1.1 ng/mL).

### Indices of Inflammation

As concerns the circulating levels of IL-6 and IL-8 in the IBS-D patients, IL-6 significantly decreased at the end of the study (5.2 ± 0.21 pg/mL vs. 4.8 ± 0.14 pg/mL; *P* = 0.025). On the contrary, the IL-8 circulating levels were not affected by the dietary treatment (4.0 ± 0.18 pg/mL vs. 4.0 ± 0.21 pg/mL).

### Intestinal Dysbiosis and Bacterial Translocation

**Figure 5:**
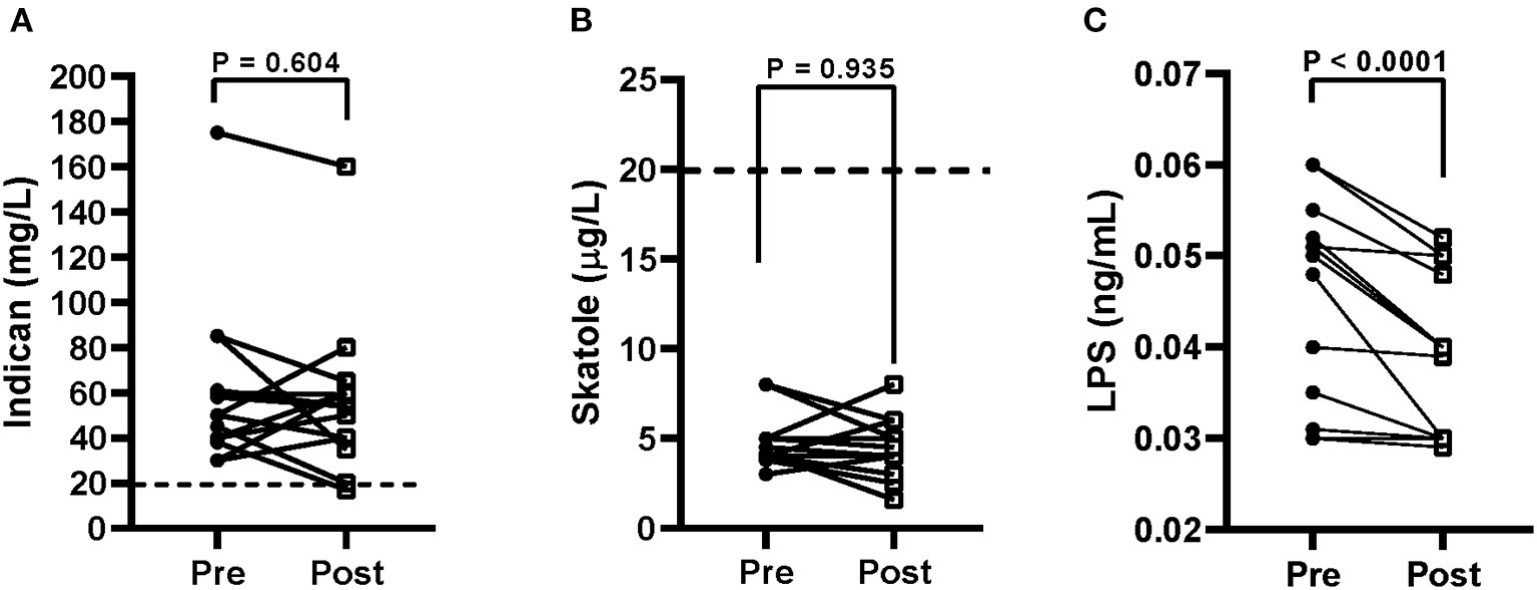
Urinary indican **(A)**, urinary skatole **(B)**, and serum lipopolysaccharide (LPS) **(C)** levels before (pre) and after (post) 12 weeks of Tritordeum-based diet in 16 IBS-D patients. Data expressed as means ± SEM. Wilcoxon rank-sum test was used to compare pre- and post-treatment data. Differences considered significant at *P* < 0.05. Dotted line indicates the grade I dysbiosis for indican (20 mg/L) and skatole (20 μg/L), respectively.

At baseline, the urinary indican concentrations in the IBS-D patients were approximately three times the level of 20 mg/L (57.9 ± 8.9 mg/L), suggesting the presence of grade I fermentative dysbiosis. At the end of the diet, a slight decrease of the urinary concentrations occurred (54.6 ± 8.1 mg/L) (Figure 5A).

The urinary skatole concentrations were below the level of 20 μg/L, and they were unaffected by diet (4.5 ± 0.37 μg/L vs. 4.5 ± 0.9 μg/L; Figure 5B). Finally, the concentrations of LPS were significantly lower (*P* < 0.0001) at the end of the diet (0.041 ± 0.002 ng/mL) than at the start of the study (0.048 ± 0.003 ng/mL), suggesting a reduction in the bacterial translocation (Figure 5C).

## Discussion

IBS is a chronic functional disorder with a still not entirely known etiology. By definition, patients suffer from alterations in bowel functions, accompanied by abdominal pain and discomfort, all symptoms recurring in the long term (29).

In the IBS pathophysiology and, more in general, in FGIDs, several mechanisms have been examined as potential factors, including the altered gut microbiota, low-grade inflammation, psychological factors, and food intolerances. Among the latter, the role of wheat consumption is still under debate (30).

In this regard, the symptom profile of IBS-D (diarrhea, bloating, or abdominal pain) is similar to that caused by NCWS, a condition where patients have symptoms that may start or be aggravated by gluten, although CD has been excluded (31). There is active discussion as to whether NCWS must be considered as a real separate disease from IBS. According to recent evidence, the spectrum of symptoms in NCWS patients may be due not only to gluten proteins but also to other wheat-related components associated with FODMAPS, mainly fructans, responsible for digestive symptoms (32).

In this framework, dietary advice has been considered one of the first treatment choices for IBS. A restriction in gas-producing vegetables and fiber intake, along with reduced coffee and tea consumption, have been proposed for several years as a strategy to mitigate IBS symptoms (33), and more recently, the FODMAP diet with avoidance of fermented carbohydrates has been proven to manage GI symptoms effectively, at least in well-defined IBS patients(6).

However, according to Böhn et al. (34), 50% of IBS patients seem not to improve their symptoms by following a low-FODMAP diet, so new dietetic options are desirable to expand nutritional strategies in the management of IBS beyond the mere therapeutic approach.

Tritordeum is a new cereal species obtained from crossing durum wheat with a wild barley species. Its composition is different from bread wheat. Firstly, it lacks the D genome (11). Therefore, this cereal does not contain the gliadin-related proteins located on it, particularly the 33-mer protein fragment of the α-gliadins, which is known to be particularly immunogenic. Secondly, amylase/trypsin inhibitors and the fructan content are slightly lower in Tritordeum than bread wheat (12).

In tested populations of healthy controls and NCWS patients, Tritordeum bread's acceptance has been similar to that of the wheat bread usually consumed and significantly higher than the gluten-free bread (11, 12, 35). Therefore, Tritordeum may be an attractive option for those who want to reduce their gluten intake.

In the present work, we performed a dietary intervention in patients with IBS-D with Tritordeum-based foods (bread, baked goods, and pasta) because we believe that patients with IBS-D represent a more appropriate population than patients with non celiac gluten intolerance. In this regard, the peculiarity of Tritordeum is not limited to the different content of gliadin but also to other components able to trigger gastrointestinal symptoms in sensitive patients. More properly, we should speak of wheat intolerance than gluten intolerance. To our knowledge, this is the first report on the possible use of these foods in the management of IBS-D.

As for our previous research on LFD in IBS-D, the efficacy of this diet was evaluated in a long-lasting period since it is undoubtedly more demonstrative of the magnitude of the effect on GI symptoms than a diet of only seven days. Besides, significant reductions in weight, BMI, abdominal and waist circumferences were observed at the end of the diet. Weight loss and BMI reduction were not among the goals of this study. However, these reductions were likely due to duration and diet restrictions. Thus, weight loss and reduced BMI should be considered a consequence of a long-term personalized diet, even if the diet's energy intake has not been significantly reduced. Despite increased protein percentages, a significant decrease in FFM occurred at the end of the Tritordeum-based diet. This reduction was the consequence of the significant decrease in TBW and ECW. Besides, PhA, an indicator of cell membranes' integrity and water distribution between intra- and extracellular compartments (36), was significantly increased. Low PhA values can represent a low cellular mass and quality and fluid overload (37).

Interestingly, our study demonstrated that a long intervention (12 weeks) with Tritordeum-based foods significantly reduced IBS-D patients' symptoms. In this pilot study, both the single item scores and the total score of the IBS-SSS questionnaire significantly decreased after 12 weeks of diet. The decision to evaluate the efficacy of the Tritordeum diet in a long-term period seemed to us undoubtedly more representative of the magnitude of effect on GI symptoms than just a 30-day diet, due to symptoms fluctuations that occur naturally in all the IBS subtypes irrespective of medications.

Based on our present results, it is conceivable that Tritordeum-based foods can improve GI symptoms by ameliorating the GI barrier function and reducing mucosal inflammation and fermentative dysbiosis. This deduction is supported by our previous work on the effect of LFD on IBS patients. In that work (6), it was evident that the reduction of the symptomatologic score was closely related to nutritional, anthropometric, and biochemical variables, considered as a whole.

Actually, changes in s-IP accompanied the improvement in the symptom profile. The La/Ma ratio significantly decreased by 41.7%. The Tritordeum-based diet also reduced fecal zonulin levels and, although not significantly, also the serum concentrations of I-FABP, thus suggesting an improvement of the epithelium integrity. Accordingly, the lower circulating levels of LPS after diet suggested a lower translocation of Gram-negative bacteria throughout the intestinal villi (38). On the contrary, the urinary indican concentrations in IBS-D patients were far above the level of 20 mg/L, suggesting the presence of altered microbiota in the small intestine. As already observed with LFD (5, 6), the diet alone could probably be inadequate to restore the balance of the microbial populations in that tract of the intestine completely.

The precise reasons why wheat or some specific components of wheat (e.g., gluten) can elicit IBS-type symptoms are still controversial. When administered endoscopically into the duodenal mucosa, wheat affected the mucosal integrity in the small intestine (39). In another study by Wu et al. (40), intestinal permeability was significantly increased after gluten challenge in gluten-sensitive IBS-D patients.

Therefore, as reported by Usai-Satta et al. (41), an incomplete degradation of gluten or other wheat proteins could consent undigested peptides to pass through an altered and more permeable mucosal barrier and aggravate GI symptoms. This mechanism could be conceivable, at least in a subset of IBS patients (42). However, based on the present results, the different composition of Tritordeum compared to wheat could restore the integrity of small intestinal mucosa, thus inhibiting the crossing of immunogenic peptides.

In IBS patients who report their symptoms linked to food, LFD appears to be a satisfying option, at least for defined periods, as already proven in our previous research (5, 6). Undoubtedly, this dietary approach, especially in its first phases, is relatively complex and challenging to follow for a lifetime, requiring close monitoring by trained nutritionists and gastroenterologists, who are not always available. For this reason, alternative, more feasible dietetic options could be preferable. In this framework, the use of Tritordeum-based foods could be acceptable and sustainable in the long period, at least for those IBS patients who complain of intolerance to foods containing wheat.

The study has some weaknesses. Firstly, the reported results derive from a pilot study, so the cohort of patients is too small to draw firm conclusions. A blinded randomized trial with common wheat-based food or Tritordeum would be more satisfactory to better evaluate Tritordeum in patients with IBS. However, the difference in taste and appearance between wheat and Tritordeum food could significantly limit the effectiveness of this study design. Secondly, the finding of a fermentative dysbiosis is not supported by data deriving from other more adequate methods since an appropriate analysis of bacterial populations in the GI tract was not performed (i.e., by molecular analysis of 16S rRNA genes). Further research is needed to investigate the still unveiled aspects linking wheat consumption and the IBS profile. Although our current results and data in the literature support this hypothesis, the actual pathophysiological mechanisms underlying it are still largely unclear.

In conclusion this preliminary report demonstrates that Tritordeum may be a viable option for general food processing and those IBS patients who experience symptoms due to the ingestion of traditional wheat and cereals. In this context, the dietary use of Tritordeum-based foods could be a promising approach for improving and supporting epithelial barrier function and integrity. This positive action on the intestinal barrier may add host protection against the invasion and translocation of pathogens by contemporary contributing to the prevention and treatment of functional GI diseases.

## Data Availability Statement

The raw data supporting the conclusions of this article will be made available by the authors, without undue reservation.

## Ethics Statement

The studies involving human participants were reviewed and approved by the Ethics Committee of IRCCS Ospedale Oncologico - Istituto Tumori Giovanni Paolo II, Bari, Italy (N. 274/C.E. 12.12.17). The patients/participants provided their written informed consent to participate in this study.

## Author Contributions

FR and GR: conceptualization. ML and BD'A: methodology. ML and FR: formal analysis and writing—original draft preparation. LP, AO, and VT: investigation. BD'A: data curation. FR, GR, and GG: writing—review and editing. FR: supervision and project administration. All authors have read and agreed to the published version of the manuscript.

## Funding

This work was supported by the Italian Ministry of Health RC 2020–2021, Prog. N. 16 (DDG n. 700/2020). Tritordeum bread, bakery products, and pasta were provided by Intini& C. SAS di Intini Bartolomeo, Putignano, Bari, Italy.

## Conflict of Interest

The authors declare that the research was conducted in the absence of any commercial or financial relationships that could be construed as a potential conflict of interest.

## Publisher's Note

All claims expressed in this article are solely those of the authors and do not necessarily represent those of their affiliated organizations, or those of the publisher, the editors and the reviewers. Any product that may be evaluated in this article, or claim that may be made by its manufacturer, is not guaranteed or endorsed by the publisher.

## Abbreviations

BIA: bioelectrical impedance analysis
BCM: body cell mass
CD: celiac disease
DAO: diamine oxidase
ECW: extra cellular water
FFM: fat-free mass
FGID: functional, non-organic gastrointestinal disorder
FM: fat mass
FODMAPs: fermentable oligosaccharides, disaccharides, monosaccharides, and polyols
GFD: gluten free diet
GI: gastrointestinal
IBS: irritable bowel syndrome
IBS-C: irritable bowel syndrome with constipation
IBS-D: irritable bowel syndrome with diarrhea
IBS-M: irritable bowel syndrome with a mixture of diarrhea and constipation
IBS-SSS: IBS symptom severity score
IDARS: IBS diet adherence report scale
I-FABP: intestinal fatty acid-binding protein
IL: interleukin
La: lactulose
LFD: low FODMAPs diet
LPS: lipopolysaccharide
Ma: mannitol
NCWS: non-celiac wheat sensitivity
PhA: phase angle
SAT: sugar absorption test
s-IP: small intestinal permeability
Su: sucrose
TBW: total body water
TJ: tight junction.
