# Corrigendum: Managing symptom profile of IBS-D patients with Tritordeum-based foods: results from a pilot study

**DOI:** 10.3389/fnut.2023.1196030

**Published:** 2023-04-14

**Authors:** Francesco Russo, Giuseppe Riezzo, Michele Linsalata, Antonella Orlando, Valeria Tutino, Laura Prospero, Benedetta D'Attoma, Gianluigi Giannelli

**Affiliations:** ^1^Laboratory of Nutritional Pathophysiology, National Institute of Gastroenterology, IRCCS “Saverio de Bellis”, Castellana Grotte, Bari, Italy; ^2^Laboratory of Nutritional Biochemistry, National Institute of Gastroenterology, IRCCS “Saverio de Bellis”, Castellana Grotte, Bari, Italy; ^3^Scientific Direction, National Institute of Gastroenterology, IRCCS “Saverio de Bellis”, Castellana Grotte, Bari, Italy

**Keywords:** diet, dysbiosis, gastrointestinal symptoms, inflammation, intestinal permeability, irritable bowel syndrome, Tritordeum

In the published article, there was an error in affiliations 1, 2, and 3 Instead of “National Institute of Gastroenterology “S. de Bellis” Research Hospital, Castellana Grotte, Italy,” the institution should be “National Institute of Gastroenterology, IRCCS “Saverio de Bellis”, Castellana Grotte, Bari, Italy”.

The authors apologize for this error and state that this does not change the scientific conclusions of the article in any way. The original article has been updated.

